# Smoking Enhances the Proinflammatory Effects of Nucleotides on Cytokine Release from Human Lung

**DOI:** 10.1371/journal.pone.0099711

**Published:** 2014-06-30

**Authors:** Kylie Belchamber, David A. Hall, Susanna M. O. Hourani

**Affiliations:** 1 Faculty of Health and Medical Sciences, University of Surrey, Guildford, Surrey, United Kingdom; 2 Fibrosis Discovery Performance Unit, GlaxoSmithKline, Stevenage, Hertfordshire, United Kingdom; University of Bern, Switzerland

## Abstract

Nucleotides have effects on immune cells which are complex but generally proinflammatory, and have been suggested to play a role in smoking-related lung diseases. However, there have been no studies directly measuring functional responses to nucleotides in human lungs taken from smokers. We used fragments of post mortem human lung from smokers and non-smokers, incubated them with a range of nucleotides (4–1000 µM) in the presence of lipopolysaccharide (LPS; 10 µg/ml) for 24 hours and measured cytokines (IL-1β, IFNγ, IL-17, TNFα, IL-6, IL-8, IL-2 and IL-10) in the supernatants using multiplex immunoassays. Although the basal cytokine levels in the smokers were generally higher in the smokers than the non-smokers, there were no significant differences in either the basal release or the LPS-stimulated release of any of the cytokines when lungs from smokers and non-smokers were compared. There were no significant effects of ATP, ADP, AMP, UTP, α,β-methylene-ATP, P^1^, P^4^-diATP, 2-methylthio-ATP or Bz-ATP on the release of cytokines from the lungs. However, the stable ATP analogue ATPγS increased the release of IL-1β and IFNγ, and the effect was greatly increased in lungs from smokers. In non-smokers but not in smokers ATPγS increased the release of IL-17. Overall these results clearly demonstrate for the first time that in normal human lung a stable ATP analogue can enhance LPS-induced pro-inflammatory cytokine release, and that these effects are greatly altered by a prior history of smoking. This provides strong support for the suggestion that nucleotides are involved in the pathogenesis of smoking-related diseases.

## Introduction

There is increasing interest in the role of nucleotides in immune and inflammatory responses, and in particular their role in lung diseases [1]. There are currently known to be eight subtypes of G protein coupled P2Y receptors (P2Y_1,2,4,6,11,12,13,14_) and seven subtypes of ionotropic P2X receptors (P2X_ 1–7_) which respond to purine and pyrimidine nucleotides [2], and nearly all of these subtypes can be found on cells in the airways [1]. It has been suggested that ATP may play a role in the pathogenesis of asthma, and allergen challenge has been shown to result in an increase in ATP in bronchoalveolar lavage fluid from asthmatic patients [3]. In a mouse model of asthma, allergen challenge also resulted in an increase in ATP concentrations in bronchoalveolar lavage fluid (BALF) and caused asthma-like symptoms which could be inhibited by administration of apyrase (which breaks down ATP) or by non-selective ATP antagonists such as suramin [3]. These findings were interpreted as indicating recruitment and activation of lung dendritic cells by ATP, resulting in induction of asthma-like responses. The P2X_7_ receptor has been suggested to be involved in this process, and P2X_7_ knockout mice display reduced airway reactivity and leukocyte recruitment [4]. The P2X_7_ receptor is known to play a key role in the processing and release of the proinflammatory cytokine IL-1β [5]–[8], and in patients with asthma P2X_7_ receptors are upregulated on eosinophils and on macrophages in bronchoalveolar lavage fluid, which secreted larger amounts of IL-1β in response to a P2X_7_ agonist [4]. Reduced P2X_7_ function was associated with a lower incidence of asthma in children at high risk of the disease [9].

Emphysema and chronic obstructive pulmonary disease (COPD) are smoking-related lung diseases in which ATP has been suggested to play a role [1], [10]. In studies in mice, exposure to cigarette smoke increased the amount of ATP in BALF, and this was associated with inflammation and emphysema. Cigarette smoke caused the release of ATP from neutrophils, an increase in ATP in BALF and upregulation of P2 receptors on neutrophils, macrophages and lung tissue [11], [12]. Both cigarette smoke and ATP caused the release of the chemokine CXCL8 and elastase (both of which are involved in emphysema and COPD) which could be prevented by suramin or apyrase [11]. Suramin also reduced the smoke-induced lung inflammation and emphysema, and genetic deletion of the P2Y_2_ receptor reduced the smoke-induced production of cytokines including IFN-γ and IL-1β and was also protective against smoke-induced inflammation [12]. Genetic deletion of the P2X_7_ receptor or a selective P2X_7_ antagonist has also been shown to reduce smoke-induced macrophage and neutrophil accumulation, release of a variety of cytokines, including IL-1β and caspase 1 activation, which is a key step in the release of IL-1β [13], [14].

In a human study comparing non-smokers, healthy smokers and patients with COPD, there was a progressive increase in these groups in ATP levels in BALF, acute smoke exposure led to a further increase in the smokers and ATP levels were negatively correlated with lung function in the COPD patients [15]. Exhaled breath condensates from patients with COPD contained higher levels of purines than those from healthy subjects, and the levels correlated with the severity of the disease [16]. In addition, enhanced responses to ATP were found in blood neutrophils and airway macrophages taken from patients with COPD, and there was upregulation of P2Y_2_ and P2X_7_ receptors respectively on these cells. In particular, there was an increase in ATP-induced IL-1β release from airway macrophages isolated from BALF taken from patients with COPD, a response mediated through activation of P2X_7_ receptors [15]. Lung tissue from patients with COPD or from smokers had higher levels of caspase 1 activity than lung tissue from non-smokers [13], which could have been a result of increased activation of the P2X_7_ receptor although this was not addressed in this study.

The studies discussed above implicate ATP as the mediator of cigarette-smoke-induced release of inflammatory mediators, and suggest that ATP plays an important role in the pathogenesis of smoking-related lung disease. However, there have been no studies directly measuring functional responses to nucleotides in lung tissue from human smokers. In the present study we have directly addressed this issue, and have compared the release of cytokines from post-mortem human lung tissue from healthy smokers and non-smokers. We used ATP itself, its stable analogues adenosine 5-0-3-thiotriphosphate (ATP-γ-S) and adenosine 5′-α,β-methylenetriphosphonate (α,β-methylene-ATP), the related nucleotides ADP, AMP and UTP, P^1^, P^4^-diadenosine tetraphosphate (P^1^, P^4^-diATP), 2-methylthioadenosine 5′-triphosphate (2-methylthio-ATP) and 2′(3′)-O-(4-benzoylbenzoyl)adenosine-5′-triphosphate (Bz-ATP) to stimulate cytokine release in the presence of lipopolysaccharide (LPS), and have shown for the first time that smoking dramatically alters the nucleotide-induced release of proinflammatory cytokines from human lung.

## Materials and Methods

The human biological samples were sourced ethically and their research use was in accord with the terms of the informed consents. Human lungs from brain dead donors, which were not suitable for transplantation, were obtained from the National Disease Research Interchange (Philadelphia), a not-for-profit tissue procurement organisation based in the USA, under the US Uniform Anatomical Gifts Act. Consent for research was obtained from the next of kin, and information received about the donor included age, sex, race, height, weight, medical history, consumption of alcohol, cigarettes and drugs, time on a ventilator, and cause of death. Tissue acquisition and the research proposal were approved internally within GSK and by NDRI (who do not specifically require IRB approval to supply tissue). Since the research we perform was within the terms of the original consent, separate ethical approval was not required for this use of the tissue. A 5 cm piece of lung parenchyma was finely chopped with scissors in a Petri dish. The suspension was then filtered through a 1 mm square sieve into a sterile Petri dish. The filtered fragments were transferred into a fresh, pre-weighed Falcon tube. Residual tissue was further chopped using scissors, and filtered once more. These fragments were added to the pre-weighed Falcon tube and made up to 50 ml with lung-tissue culture medium (RPMI without phenol red (GIBCO), containing 0.1% BSA, 100 U/ml penicillin, 2 mM glutamine, 100 µg/ml streptomycin). Fragments were centrifuged at 200 g for 2 minutes, 5 times to remove blood, followed by a final spin of 300 g for 5 minutes. The supernatant was discarded and the pellet weighed. Fragments were plated at 0.04 g/well in 700 µl lung tissue culture medium in a 48 well tissue culture plate, with care taken to ensure all wells had approximately the same amount of tissue. Nucleotides (1.4 µl, 4 to 1000 µµ final concentration) and/or LPS (7 µl, 10 µg/ml final concentration) were then added and plates incubated for 24 hours at 37°C in a 5% CO_2_ atmosphere. After 24 hours, 200 µl supernatant was removed from wells, and stored at −80°C.

Cytokine levels in supernatants were analysed using MSD cytokine multiplex immunoassays (Mesoscale), according to manufacturer’s instructions. The cytokines measured were IL-1β, TNFα, IFNγ, IL-17, IL-6, IL-8, IL-2 and IL-10. In brief, samples were defrosted to room temperature and 25 µl was added to the appropriate well of the MSD plate, and incubated at room temperature with vigorous shaking for 2 hours. 25 µl of the appropriate detection antibody was added to each well, and plates incubated for 1 hour at room temperature with vigorous shaking. Plates were washed using an automatic plate washer, and 150 µl per well of 2x MSD Read Buffer diluted in distilled water was added to the plates. Plates were read within 30 minutes of the addition of Read Buffer on the MSD SECTOR instrument.

### Materials

ATP disodium salt, ATPγS tetralithium salt, α,β-methylene-ATP lithium salt, ADP disodium salt, AMP disodium salt, UTP trisodium salt, 2-methylthio ATP tetrasodium salt, P^1^, P^4^-diATP ammonium salt and BzATP triethylammonium salt (Sigma Aldrich) were made up as 500 mM stock concentrations stored at −4°C, then diluted further using distilled water. LPS (Sigma Aldrich) was prepared as a stock concentration of 1 mg/ml with sterile PBS and stored at −4°C.

### Statistical Analysis

The raw data from all studies were analysed for statistical significance using Graphpad Prism software (Graphpad Software Inc.). In order to compare the effect of LPS against the untreated control, a paired two-tailed t-test was used, and to compare the basal or the LPS-stimulated cytokine levels in smokers and non-smokers unpaired t tests were used. A one way repeated measures ANOVA with *post-hoc* Dunnett’s test was used to compare the effect of nucleotide in the presence of LPS with LPS alone. These results were shown as a percentage of the effect of LPS alone in the graphs in order to reduce the variation between donors. Although the responses to each nucleotide was analysed separately, multiple nucleotides have been shown on the same graph for ease of comparison. For comparison of the effects of ATPγS in smokers and non-smokers, two-factor ANOVA was used (for the factors concentration and smoking), with Bonferonni post-hoc tests.

## Results

**Figure 1:**
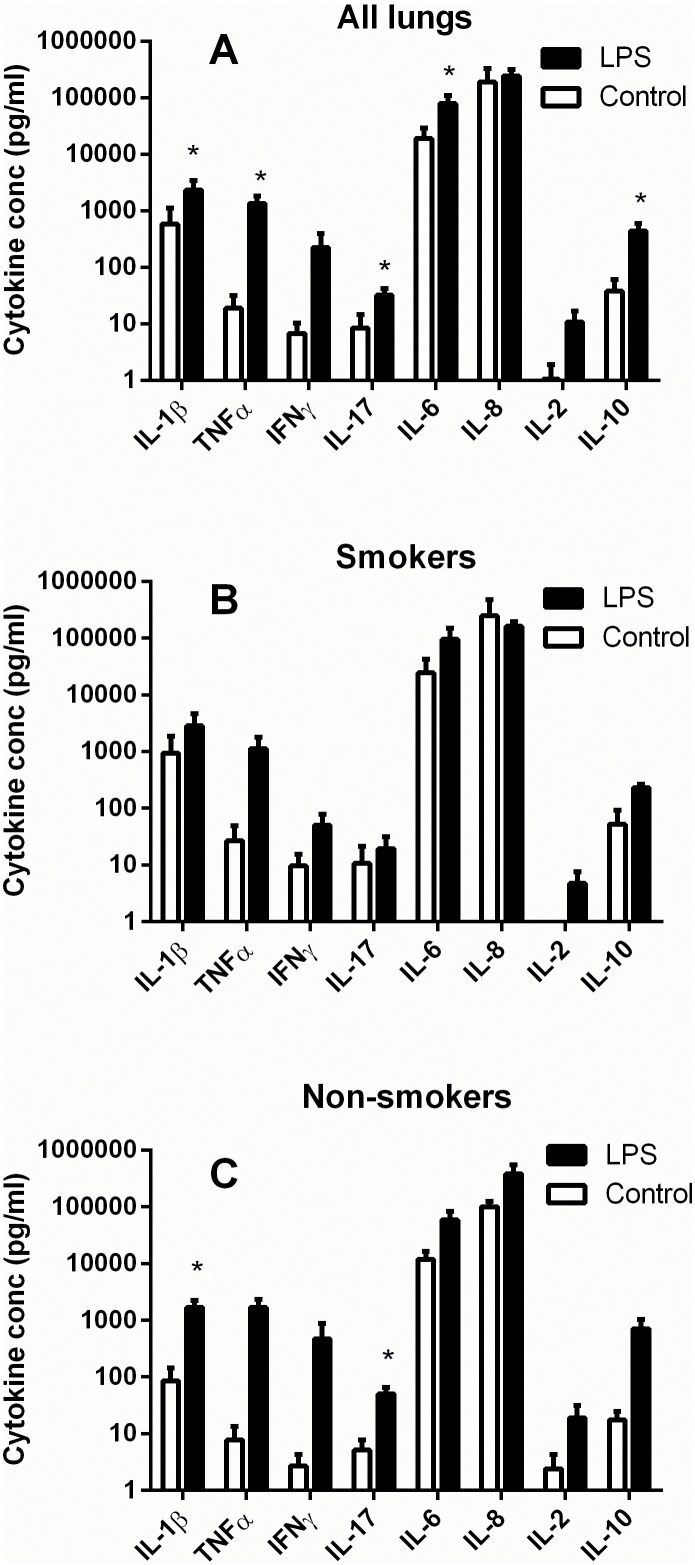
Effect of LPS on release of cytokines from human lung fragments. Post-mortem human lung parenchyma was chopped and sieved to obtain pieces smaller than 1 mm and incubated for 24 h at 37°C in the presence (black bars) or absence (white bars) of LPS (10 µg/ml). The supernatants were then analysed for the cytokines shown by MSD multiplex immunoassays. A shows the results from all the lungs (n = 7–12), while B and C show results from smokers (n = 4–7) and non-smokers (n-3–5) respectively. Values are mean ± s.e.mean, * indicates a significant difference between control and LPS-stimulated values, p<0.05, paired t test.

In each of the lungs LPS (10 µg/ml) caused increases in the release of IL-1β, TNFα, IFNγ, IL-17, IL-6, IL-8, IL-2 and IL-10 but the magnitude of the responses varied greatly between donors and the effects did not achieve statistical significance for IFNγ, IL-8 or IL-2 (Figure 1A, paired t test). When the donors were separated into smokers and non-smokers, the response to LPS in the non-smokers only achieved statistical significance for IL-1β and IL-17 (Fig. 1C; p<0.05, paired t test). In lungs from the smokers LPS (10 µg/ml) did not cause a statistically significant increase in release of any of the cytokines (Fig. 1B; p>0.05, paired t test). Although the basal cytokine levels in the smokers were generally higher in the smokers than the non-smokers, there were no significant differences in either the basal release or the LPS-stimulated release of any of the cytokines when lungs from smokers and non-smokers were compared (p>0.05, unpaired t-test).

**Figure 2:**
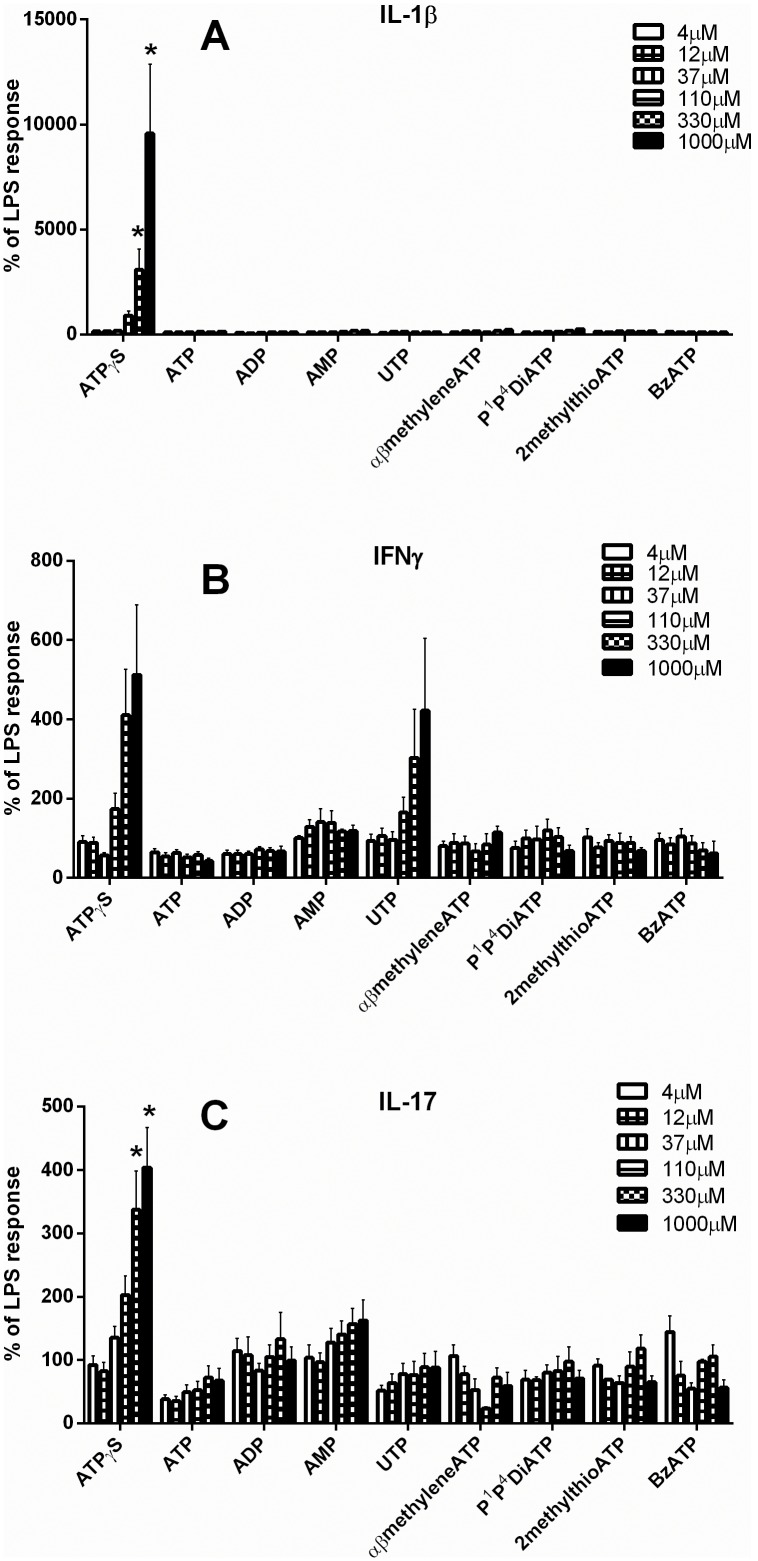
Effect of nucleotides on release of cytokines from human lung fragments in the presence of LPS. Post-mortem human lung parenchyma was chopped and sieved to obtain pieces smaller than 1 mm and incubated for 24 h at 37°C in the presence of LPS (10 µg/ml) alone or in the presence of a range of nucleotides (ATPγS, ATP, ADP, AMP, UTP, α,β-methylene-ATP, P^1^, P^4^-diadenosine tetraphosphate, 2-methylthio-ATP, Bz-ATP, 4–1000 µM). The supernatants were then analysed for cytokines by MSD multiplex immunoassays. A, B and C show the results for IL-1β, IFNγ and IL-17, n = 3–18, displayed as % of the release caused by LPS alone, mean ± s.e.mean. * indicates a significant difference between the release in the presence of the nucleotide and that to LPS alone (one-way repeated measures ANOVA with Dunnett’s post-hoc test) carried out on the data in pg/ml.

When a range of nucleotides were tested for their ability to affect the LPS-stimulated response, ATPγS stimulated the release of IL-1β and IL-17, an effect which achieved statistical significance at a concentration of 330 and 1000 µM (p<0.05, one-way ANOVA), and there was an apparent concentration-dependent increase in the release of IFNγ in response to ATPγS and UTP which did not achieve statistical significance and where the individual variation was very large (Figure 2). There was no significant effect of any other nucleotide (ATP, ADP, AMP, UTP, α,β-methylene-ATP, P^1^, P^4^-diATP, 2-methylthio-ATP, Bz-ATP) on any other cytokine measured (TNFα, IL-6, IL-8, IL-2 and IL-10; results not shown).

**Figure 3:**
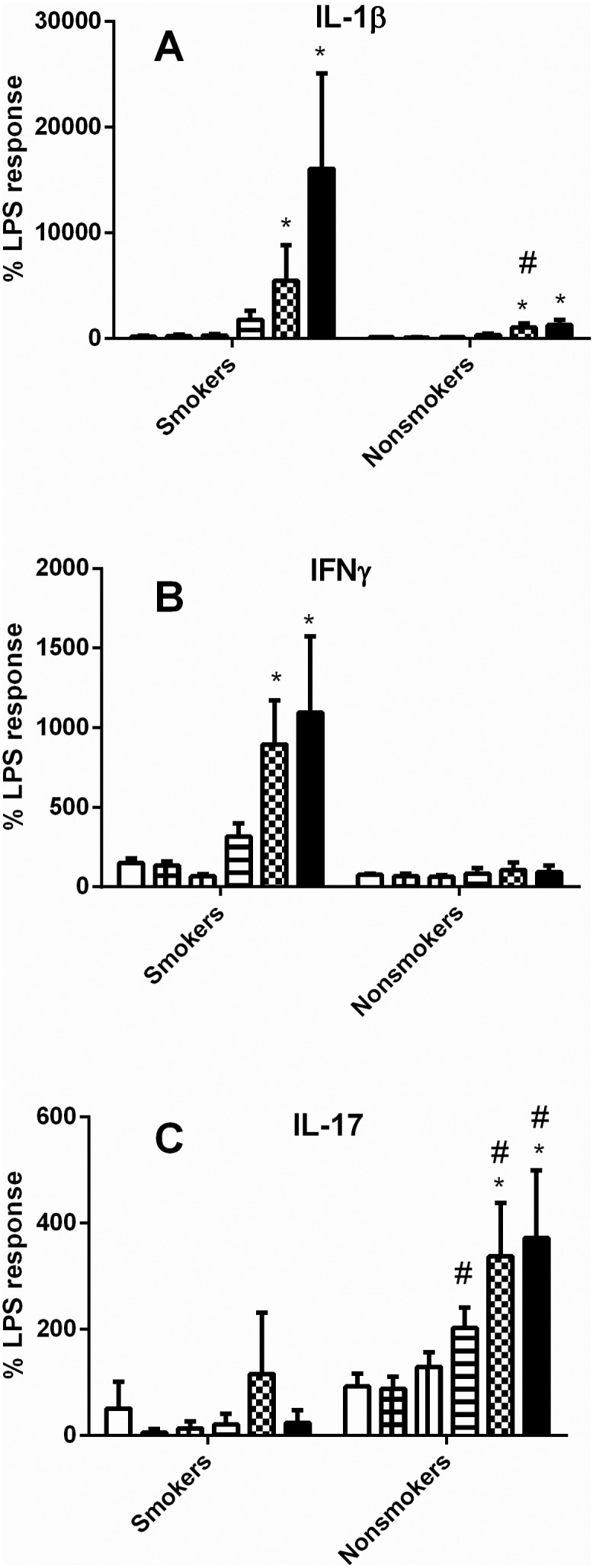
Effect of ATPγS on release of cytokines from human lung fragments from smokers and non-smokers in the presence of LPS. Post-mortem human lung parenchyma was chopped and sieved to obtain pieces smaller than 1 mm and incubated for 24 h at 37°C in the presence of LPS (10 µg/ml) alone or in the presence of ATPγS (4–1000 µM). The supernatants were then analysed for cytokines by MSD multiplex immunoassays. A, B and C show the results for IL-1β, IFNγ and IL-17, n = 3, displayed as % of the release caused by LPS alone, mean ± s.e.mean. * indicates a significant difference between the release in the presence of ATPγS and that to LPS alone (one-way repeated measures ANOVA with Dunnett’s post-hoc test), while # indicates a significance difference between the release in the smokers and the non-smokers (two-factor ANOVA with Bonferonni post-hoc test), carried out on the data in pg/ml.

When the lungs from smokers and non-smokers were compared, ATPγS significantly enhanced the effect of LPS on the release of IL-1β in both types of lung at 330 and 1000 µM (one-way ANOVA), but the effect was significantly greater in the smokers (Figure 3A; two factor ANOVA). Similarly, for IFNγ the effect of ATPγS was much greater in the smokers and achieved statistical significance at 330 and 1000 µM (one-way ANOVA), while there was no effect at all in the non-smokers (Figure 3B). There was no significant effect of UTP on release of IFNγ in either type of lung (results not shown). For IL-17 the situation was the opposite, with ATPγS causing a concentration-dependent enhancement of the effect of LPS in the non-smokers which achieved significance at 330 and 1000 µM (one-way ANOVA), while there was no effect in the smokers, and there was a significant difference in the responses of the two types of lung (two factor ANOVA, Figure 3C).

## Discussion

The results presented here clearly show that smoking greatly increases the release of IL-1β and IFNγ from human lungs in response to the non-hydrolysable ATP analogue, ATPγS, while reducing the release of IL-17. The basal levels of cytokine release were not significantly affected by smoking, and neither was the release in the presence of LPS, suggesting that smoking selectively affects the responses to nucleotides rather than acting to alter cytokine release non-specifically.

The tissues used in this study were small fragments of post-mortem human lungs which had been washed free of blood cells but which still contained a number of different cell types, including epithelial cells, connective tissue and resident immune cells including monocytes, macrophages, dendritic cells, lymphocytes including natural killer (NK) cells and granulocytes [17]. There is some evidence to suggest that many of these cells occur in larger numbers in the lungs of smokers but that their function may be compromised or altered [18]. In pilot studies nucleotides alone had no effect on cytokine release, so LPS was used to prime the lung fragments. LPS, a bacterial endotoxin, is known to act via the Toll-like receptor TLR4, which recognises pathogen-associated molecular patterns and triggers a widespread immune response including the release of pro-inflammatory cytokines [19], [20]. As expected, LPS alone increased the release of proinflammatory cytokines including IL-1β from the lung fragments, and we tested the effects of nucleotides in the presence of LPS. We used a range of nucleotides in an attempt to activate the major receptors likely to mediate the responses and to give some clues as the receptor type(s) involved. ATP was used as the endogenous agonist at all the P2X receptors as well as at the P2Y_1_, P2Y_2_, P2Y_11_ receptors, while UTP was used as the endogenous agonist at P2Y_2_ and P2Y_4_, and ADP as the endogenous agonist at P2Y_1_, P2Y_12_ and P2Y_13_ [21]–[23]. ATPγS was used as an analogue of ATP which is resistant to enzymatic degradation but retains activity at all the receptors on which ATP is active [23]. α,β-methylene ATP was used as another stable analogue of ATP which has some selectivity for P2X_1_ and P2X_ 3_ receptors but has also been shown to as an agonist at P2Y_11_ receptors [24], P^1^, P^2^-DiATP is active at P2Y_2_ and P2X_1_ receptors, 2-methylthio-ATP has some selectivity for P2Y_1_, P2Y_12_ and P2X_1_ receptors while BzATP is more potent than ATP at the P2X_7_ receptor [21], [23]. Rather than using adenosine (which is rather insoluble in water) to activate any adenosine receptors, we used AMP which is inactive at P2 receptors but rapidly breaks down to adenosine [23].

Of the compounds tested only ATPγS had any significant effect on cytokine release, and it enhanced the release of IL-1β, IFNγ and IL-17 but not of the other cytokines measured. ATP is rapidly degraded by dephosphorylation, ultimately to adenosine which can be taken up or further metabolised by ectonucleotidases which are present on the surface of all cells and known to play important roles in immune function [25], [26]. Analogues of ATP which have modifications to the phosphate chain, such as ATPγS and α,β-methylene ATP, are degraded much more slowly or not at all by these enzymes while analogues with an unmodified phosphate chain, such as 2-methylthio-ATP, are rapidly broken down [27], [28]. In the experiments reported here the nucleotides were incubated with the lung fragments for 24 hours at 37°C, to allow time for the synthesis and release of the cytokines, so the lack of effect of most of the nucleotides tested may be due to their breakdown. Indeed, degradation is well known to reduce greatly the potency of susceptible analogues in many tissues, and to distort observed structure-activity relationships [28]–[30]. The lack of effect of α,β-methylene ATP however cannot be explained by degradation of this compound, but instead indicates that P2X_1_, P2X_3_ receptors and P2Y_11_ receptors are unlikely to be involved in the modulation of cytokine release [21], [23], [24]. Apart from the P2X_7_ receptor, P2X receptors are not thought to regulate cytokine production, but P2Y_11_ receptors on dendritic cells in particular have been reported to have complex effects on cytokine production [31]. The lack of effect of AMP suggests that adenosine receptors are not important here in modulating cytokine release although it has been shown to have effects on immune cells which are generally inhibitory [32].

P2X_7_ receptors are known to play a key role in the release of IL-1β from immune cells including macrophages [5]–[7], and it is likely that this is the receptor responsible here for the increased IL-1β release seen with ATPγS in both smokers and non-smokers, although this can only be a preliminary conclusion in the absence of data on the effect of a selective P2X_7_ antagonist on this response. The large increase in release seen here in the lungs from smokers is consistent with increased ATP-induced IL-1β release from immune cells taken from smokers [15], and studies in mice suggesting a role of ATP and the P2X_7_ receptor in smoke-induced pro-inflammatory lung responses [11], [12], [14]. These studies reported that smoking caused an upregulation of P2 receptors, and this may explain the greatly enhanced responses that we observed. Smoke inhalation has been reported to alter the activity of ectonucleotidases on blood platelets in rats [33], and nicotine itself inhibited ectonucleotidase activity in rat lymphocytes [34]. However, changes in ectonucleotidase activity in the lungs of smokers could not account for the differences that we observed in the responses to the stable analogue ATPγS between smokers and non-smokers.

IFNγ is a pro-inflammatory cytokine which is the characteristic product of Th1 lymphocytes [35], [36] but is also released by CD8^+^ (cytotoxic) T cells and a number of innate immune cells such as NK cells and professional antigen presenting cells [37], [38]. Under the conditions of our experiments it is more likely that this cytokine was released as part of an innate rather than an adaptive immune response. Among the numerous functions of IFNγ in the immune system is the activation of macrophages and priming of their response to LPS [see for example 38,39,40]. Increased production of IL-1β in response to LPS combined with IFNγ has also been reported from neutrophils [41]. In the present study, ATPγS increased the release of IFNγ from the smokers’ tissue but not from that of non-smokers, a similar effect to that seen on IL-1β release. It is not clear from our experiments whether these effects of ATPγS are related or independent. Caspase-1, which is activated by P2X_7_ receptor stimulation, is involved in the release of both IL-1β and IFNγ [42], [43] so it is possible that the release of these two cytokines is coupled. However, given the reported synergistic effects of LPS and IFNγ, it is tempting to speculate that the increased release of IFNγ drives the greater production of IL-1β particularly as there was a rather smaller, but still statistically significant, increase in IL-1β secretion in response to ATPγS in the non-smoker lung tissue in which IFNγ production was not increased. In contrast to the results presented here, ATP (or ATPγS) has been reported to inhibit IFNγ production from a number of its source innate immune cells by an action on P2Y_11_ receptors [43]. This may indicate that the effect of smoking is to modify the response of the IFNγ secreting cells to purine nucleotides, by upregulating P2X_7_ receptors but not P2Y_11_ receptors, shifting the response to a more pro-inflammatory phenotype. It should be noted here that rodents do not appear to possess a P2Y_11_ receptor gene [22], so studies on smoke inhalation in mice [eg 11,12,14] may not necessarily translate to humans.

IL-17 is a pro-inflammatory cytokine, produced by T_H_17 lymphocytes, which has been implicated in the pathogenesis of lung disease, including smoking-related emphysema [44]–[46]. Unlike IL-1β and IFNγ the release of IL-17 was enhanced by ATPγS in the non-smokers but not in the smokers, which may reflect the complex effects of nucleotides on cytokine release, which can vary depending on the concentration of agonist (eg LPS) used to stimulate release, as well as the concentration and type of cytokine involved [31].

Overall the results presented here clearly demonstrate for the first time that in normal human lung a stable ATP analogue can enhance LPS-induced pro-inflammatory cytokine release, and that these effects are greatly altered by a prior history of smoking. Further work is needed to determine the receptor type(s) involved in this response and the target cells, but the findings provide supportive evidence for a role of purinergic mechanisms in smoking-induced lung injury.
